# Tumor Killing by CD4^+^ T Cells Is Mediated *via* Induction of Inducible Nitric Oxide Synthase-Dependent Macrophage Cytotoxicity

**DOI:** 10.3389/fimmu.2018.01684

**Published:** 2018-07-23

**Authors:** Marte Fauskanger, Ole Audun Werner Haabeth, Frode Miltzow Skjeldal, Bjarne Bogen, Anders Aune Tveita

**Affiliations:** ^1^Department of Immunology and Transfusion Medicine, Oslo University Hospital, Oslo, Norway; ^2^Department of Biosciences, University of Oslo, Oslo, Norway; ^3^KG Jebsen Centre for Influenza Vaccine Research, Institute of Clinical Medicine, Faculty of Medicine, University of Oslo, Oslo, Norway

**Keywords:** macrophage, CD4^+^ T cell, immunotherapy, myeloma, nitric oxide

## Abstract

CD4^+^ T cells can induce potent anti-tumor immune responses. Due to the lack of MHC class II expression in most cancer cells, antigen recognition occurs indirectly *via* uptake and presentation on tumor-infiltrating antigen-presenting cells (APCs). Activation of the APCs can induce tumor rejection, but the mechanisms underlying tumor killing by such cells have not been established. To elucidate the molecular basis of CD4^+^ T-cell-mediated tumor rejection, we utilized a murine model of multiple myeloma, in which the T cells recognize a secreted tumor neoantigen. Our findings demonstrate that T cell recognition triggers inducible nitric oxide synthase activity within tumor-infiltrating macrophages. Diffusion of nitric oxide into surrounding tumor cells results in intracellular accumulation of toxic secondary oxidants, notably peroxynitrite. This results in tumor cell apoptosis through activation of the mitochondrial pathway. We find that this mode of cytotoxicity has strict spatial limitations, and is restricted to the immediate surroundings of the activated macrophage, thus limiting bystander killing. These findings provide a molecular basis for macrophage-mediated anti-tumor immune responses orchestrated by CD4^+^ T cells. Since macrophages are abundant in most solid tumors, evoking the secretion of nitric oxide by such cells may represent a potent therapeutic strategy.

## Introduction

Macrophages are abundantly present in most solid tumors, and are known to produce a number of factors that may greatly influence tumor growth. In most cases, tumor-infiltrating macrophages (TAMs) display M2-like phenotypic characteristics, associated with angiogenesis and tumor expansion (1). By contrast, in the context of acute inflammatory responses, macrophages may undergo classic (M1) activation, and attain cytotoxic properties. *In vivo*, such activation can be induced by cognate interaction with Th1-polarized CD4^+^ T cells (2).

Efficient anti-tumor immune responses mediated by tumor-specific CD4^+^ T cells have been reported by several groups (3–8), although in most instances, the underlying effector mechanisms remain poorly understood. Some tumor cells express MHC class II, and in such cases, cytotoxic CD4^+^ T cells have been shown to mediate direct cytotoxic effects *via* the Fas/Fas ligand (9) or perforin/granzyme pathway (3). For other tumor cell types, including the MOPC315 plasmacytoma cell line used in the present study, the tumor cells do not themselves express MHC class II, even in the presence of interferon gamma (IFN-γ) (2, 10, 11). The tumor cells are therefore unable to directly interact with tumor-infiltrating T cells (2), and antigen presentation is dependent on uptake in host antigen-presenting cells (APCs) (12). Hence, CD4^+^ T cell recognition of tumor antigen occurs in an indirect manner (2, 10, 12, 13).

We have previously demonstrated that CD4^+^ T cells reactive against a secreted myeloma protein tumor antigen can mediate protection against tumor development upon challenge with MOPC315 myeloma cells (2, 6, 7, 12). Immunoprotection occurs *via* T-cell-mediated activation and M1 polarization of TAMs, rendering them cytotoxic to neighboring tumor cells (2, 13). Such indirect tumor antigen recognition results in a change in the cytokine profile of the tumor microenvironment toward a Th1-type inflammatory response (13). Despite these observations, the molecular mechanism(s) underlying macrophage-mediated killing of tumor cells is not known.

We have here performed an in-depth characterization of macrophage-mediated cytotoxicity against MOPC315. Our results demonstrate that activated macrophages rapidly induce apoptosis in tumor cells *via* the mitochondrial pathway. This occurs in a cell contact-independent, but spatially limited fashion. Cytotoxicity is dependent on short-lived factors, and is completely abrogated in the absence of inducible nitric oxide synthase (iNOS) in TAMs. Further assays reveal a critical role of peroxynitrite formed within the tumor cells, pointing to short-lived reactive nitrogen species (RNS) as mediators of macrophage cytotoxicity.

## Materials and Methods

### Reagents, Cells, and Viral Transduction

Apocynin, taurine, and superoxide dismutase (SOD) (Sigma-Aldrich, St. Louis, MO, USA). Manganese (III) meso-tetrakis(*N*-ethylpyridinium-2-yl)porphyrin (MnTE-2-PyP; Frontier Scientific, Logan, UT, USA). L-NG-monomethylarginine monoacetate (L-NMMA; Enzo Life Sciences, Farmingdale, NY, USA). The mouse T-lymphoma cell line L5178Y and the FasL-transfected derivative FasL/L5178Y (14) were generously provided by Dr. Hideo Yagita, Juntendo University School of Medicine, Tokyo. Cells were propagated in RPMI1640-GlutaMAX medium (Sigma-Aldrich, St. Louis, MO, USA) supplemented with 10% fetal bovine serum, penicillin, and streptomycin. MOPC315 (IgA, λ2) is a transplantable BALB/c plasmacytoma obtained from the American Type Culture Collection (ATCC, Manassas, VA, USA). The present experiments utilized an *in vivo*-passaged variant, MOPC315.4, generated as previously described (15). Cell identity was regularly confirmed by intracellular flow cytometry using an idiotype-specific mAb (Ab2.1–4) (7). Retroviral transduction was performed by transfection of Phoenix-Eco cells (ATCC) with the relevant pMSCV target plasmid. Virus supernatant was harvested after 48 h. MOPC315 cells were transduced by 1 h centrifugation (1,200 × *g*) at 37°C in Retronectin-coated 24-well plates (Takara Bio, Mountain View, CA, USA). Generation of MOPC315 cells stably expressing the fluorescent proteins mCherry or green fluorescent protein (GFP) has been previously described (16). The IgA non-producing variant MOPC315.36 was kindly provided by Alexander Marks, University of Toronto. Bicistronic pMSCV-IRES-EGFP retroviral vectors encoding the apoptosis-related proteins Bcl-2, Bcl-XL, FLIP-L, and cytokine response modifier A (CrmA) (17) were generously provided by Dr. A Grandien, Karolinska Institute, Stockholm, Sweden.

### Mice and *In Vivo* Experiments

DO11.10, CByJ.129P2(B6)-Nos2^tm1Lau^/J and wild-type (WT) BALB/c mice were obtained from Jackson (The Jackson laboratory, Bar Harbor, ME, USA). Homozygous Id-specific T cell receptor-transgenic (TCR-Tg) BALB/c mice have been previously described (18). Heterozygous TCR-Tg SCID mice (6) and SCID littermates were kept on a BALB/c background. TCR-transgenic BALB/c SCID and BALB/c Rag^−/−^ mice hemizygous for the TCR transgenes were bred in-house. Offspring (50% transgenic, 50% non-transgenic) were typed by staining of blood CD4^+^ lymphocytes using the TCR clonotype-specific mAb GB113 (18). All mice were bred and maintained under special pathogen-free conditions. All experiments were approved by the Norwegian Animal Research Authority (Mattilsynet), and performed in accordance with institutional and Federation of European Laboratory Animal Science Associations guidelines.

Tumor challenge experiments were performed by subcutaneous (s.c.) injection of 1.6 × 10^5^ MOPC315 cells dissolved in 100 µL PBS. For some experiments, cells were embedded in 250 µL Matrigel to form a tumor bed of defined size, as previously described (13). Tumor development was followed by palpation and digital caliper measurement, and mice were euthanized upon developing tumors with largest diameter >10 mm. Isolation of cells from explanted Matrigels was performed as previously described (13). For adoptive transfer, mice were sub-lethally irradiated (500 cGy) at day −2, injected i.v. with 2 × 10^6^ naïve Id-specific T cells at day −1 and subjected to tumor challenge 24 h later. For *in vivo* macrophage depletion, 200 µg of anti-CCL2 mAb (clone 2H5, BioXCell, West Lebanon, NH, USA) or polyclonal hamster IgG (isotype control, BioXcell) was injected every second day for the duration of the experiment.

### Macrophage Cytotoxicity Assays

Macrophage cytotoxicity assays were performed using peritoneal macrophages obtained by lavage, or using MACS-separated CD11b^+^ cells isolated from tumors. IFN-γ/LPS activation of macrophages was performed by 4 h incubation with recombinant mouse IFN-γ (20 U/mL; Peprotech, Rocky Hill, NJ, USA), followed by addition of *Escherichia coli* 0111:B4 LPS (100 ng/mL; Sigma-Aldrich) and 20 h incubation before being used in further experiments. In some assays, macrophages were fixed by 1 min incubation in 1% formaldehyde or 0.05% glutaraldehyde in PBS on ice and washed extensively. For experiments involving macrophages and Id-specific CD4^+^ T cells, macrophage:T cell ratios of 10:1 were used, based on data from previous reports (16). Growth inhibition assays were performed by co-culture macrophages and tumor cells, at the indicated ratios, for various amounts of time. Tumor cells were then transferred to new wells, and ^3^H-thymidine was added for 18 h before harvesting. Growth was expressed as percentage counts per minute (cpm) of tumor cells cultured alone. For transwell assays, macrophages were seeded in 24-well plates, and tumor cells added in 250 µL medium in culture chamber inserts with 0.4 µm pore size (Corning Inc., Corning, NY, USA). Tumor cells were harvested after 24 h and analyzed by flow cytometry as specified below.

### *In Vitro* Imaging Assays

Macrophages and tumor cells were labeled with the fluorescent cell labeling solutions Vybrant DiI and CellTracker Violet (Life Technologies), respectively, by 30 min incubation at 37°C according to the supplied instructions. Macrophages were plated on IbiTreat chamber slides (Ibidi, Martinsried, Germany) with culture inserts. Inserts were then removed, and the slides overlaid with tumor cells in 50 µL Matrigel. The position of individual tumor cells relative to the macrophage border was determined using ImageJ v.1.47 (NIH), and frequency distribution plotted to reflect the spatial distribution of tumor cells relative to the macrophage monolayer.

Live imaging experiments were carried out on an Olympus FV 1000 confocal microscope equipped with a 20× Olympus Plan Achromat Objective, 0.4 NA, 1.2 mm WD objective. The cells were then imaged for 24 h at 37°C with 5% CO_2_, with image acquisition every 10 min. In other experiments, an IncuCyte wound maker (Essen BioScience, Ann Arbor, MI, USA) was utilized to remove a defined portion of macrophage monolayers in 96-well plates, and growth of tumor cells in the scratch area of individual wells was imaged with 20 min intervals for 48 h using IncuCyte Zoom live-cell imaging system (Essen BioScience).

### Immunofluorescence Staining

Material for cryosections was prepared by embedding in sucrose compound (O.C.T.) and stored at −80°C. Four-micrometer sections were prepared on Superfrost Plus Slides (Thermo Fischer, Waltham, MA, USA). Cytospin preparations were fixed for 30 s in ice-cold acetone prior to staining. Sections and cytospin slides were washed in PBS and incubated for 30 min in blocking buffer (0.5% BSA in PBS). Slides were then incubated with primary antibody in blocking buffer for 2 h at RT and with secondary antibody for 1 h at RT. TUNEL staining was performed using the Fluorescein *In Situ* Death Detection Kit (Roche Applied Science). Briefly, Matrigel cryosections were fixed for 20 min in 4% paraformaldehyde in PBS at room temperature, washed, and incubated for 2 min in permeabilization buffer (0.1% Triton-X100 and 0.1% sodium citrate in PBS). Cells were then incubated with TUNEL reaction mixture for 1 h at 37°C, washed and stained with anti-CD138 antibody as specified above. Sections incubated with 100 U/mL DNase I (Sigma-Aldrich) for 10 min prior to TUNEL staining were included as a positive control.

### Flow Cytometry

Staining for flow cytometry was performed by 30 min incubation with the relevant antibody in blocking buffer (PBS with 0.5% BSA). For intracellular nitrotyrosine staining, cells were fixed and permeabilized by incubation in Cytofix Fix/Perm buffer (BD Bioscience) for 20 min at 4°C. Staining was performed in Cytofix Perm/Wash buffer containing 0.5% BSA. DAF-FM diacetate staining was performed according to the manufacturer’s protocol (Life Technologies). Briefly, cells were incubated with DAF-FM diacetate for 60 min, washed and incubated in normal medium for 30 min. The cells were then stained with fluorescently labeled surface markers and analyzed by flow cytometry. Detection of activated caspase 3/7 was performed by using the CellEvent Caspase 3/7 Green detection reagent (Life Technologies) according to the manufacturer’s protocol.

### Statistical Analysis

Mann–Whitney *U* test was used for statistical analysis unless otherwise stated. For tumor challenge experiments, survival was analyzed using the log-rank test. Statistical analysis was performed using Prism 5.0 software (GraphPad Software, La Jolla, CA, USA). *p* < 0.05 was considered statistically significant. Designations *, **, and *** in figures indicate *p*-values of <0.05, <0.01, and <0.001, respectively.

## Results

### Killing of Tumor Cells by Activated Macrophages Occurs *via* Induction of the Intrinsic Apoptotic Pathway

**Figure 1:**
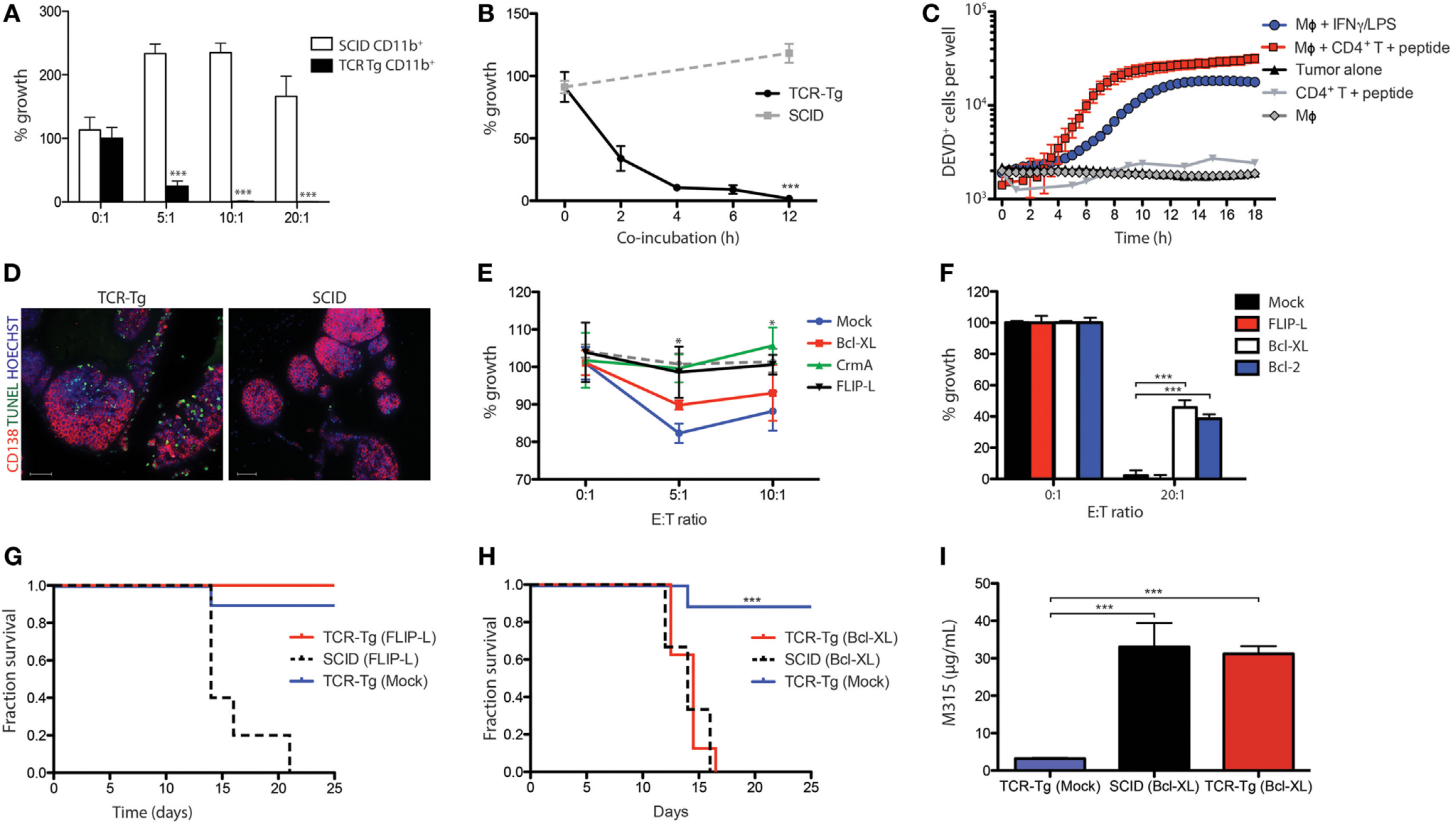
Th1/M1 macrophage-mediated killing of myeloma involves the intrinsic apoptotic pathway. **(A)** Tumor cell growth inhibition assay showing the growth of tumor cells co-incubated for 24 h with Matrigel-derived CD11b^+^ cells isolated from T cell receptor-transgenic (TCR-Tg) (black bars) or SCID (white bars) mice at day +12 following tumor challenge at the indicated ratios. Cells were cultured for 48 h (*n* = 4 per treatment condition), with the addition of ^3^H-thymidine for the last 18 h of culture. Growth was expressed as percentage counts per minute (cpm) of tumor cells cultured alone. *** signifies *p* < 0.001. **(B)** Time-dependence of macrophage-mediated cytotoxicity assayed in a tumor cell growth inhibition assay. CD11b^+^ cells were isolated from the Matrigel tumor bed at day +12 following tumor challenge in either TCR-Tg SCID or SCID mice, and co-incubated *in vitro* with tumor cells at a 20:1 ratio for the indicated time periods. The non-adherent tumor cells were then isolated, washed, and cultured for 48 h, with addition of ^3^H-thymidine for the last 18 h of culture. Growth was expressed as percentage of tumor cells cultured alone (mean ± SD). **(C)** Co-incubation assays showing accumulation of the cleaved form of the caspase 3/7 substrate DEVD within tumor cells incubated in the presence of macrophages (Mϕ), Id-specific CD4^+^ T cells, and synthetic Id peptide, as indicated. **(D)** TUNEL staining (green) of cryosections from Matrigel isolated from TCR-Tg and SCID mice at day +10 following subcutaneous challenge with 1.6 × 10^5^ MOPC315. Sections were counterstained with anti-CD138 (red) and Hoechst (blue). Images were taken using identical exposure settings. Images are representative of three independent assays with six replicates per group. **(E)** Cytotoxicity assay showing the effect of the presence of irradiated (30 Gy) FasL-expressing T-lymphoma cell line FasL/L5178Y on the growth of MOPC315 cells transduced with pMSCV-IRES-EGFP (Mock) and MOPC315 variants overexpressing Bcl-XL, cytokine response modifier A (CrmA) or FLIP-L. Mock-transfected cells co-incubated with the wild-type L5178Y variant are represented by the dotted line. Cells were co-incubated for 48 h at the indicated ratios (*n* = 8 per treatment condition), with addition of ^3^H-thymidine for the last 18 h of culture. Growth was expressed as percentage cpm of tumor cells cultured alone for the statistical difference of CrmA- and FLIP-L-overexpressing cells relative to control (Mock). **(F)** Impact of overexpression of FLIP-L, Bcl-XL, or Bcl-2 in tumor cells on macrophage cytotoxicity. Controls were transduced with the pMSCV-IRES-EGFP vector alone (Mock). Tumor cells were cultured alone or co-incubated with interferon gamma (IFN-γ)/LPS-activated macrophages at a 20:1 for 6 h, washed and cultured for 24 h, with addition of ^3^H-thymidine for the last 18 h of culture. **(G,H)** Survival curves for either Id-specific TCR-Tg or SCID mice challenge with 1.6 × 10^5^ tumor cells (*n* = 6 per group) overexpressing FLIP-L **(G)** or Bcl-XL **(H)**. **(I)** Average serum M315 levels in mice from the experiment shown in **(H)** at the time of tumor detection (day 12–16; *n* = 6 per group).

CD4^+^ T cells of idiotope (Id)-specific TCR-Tg mice recognize a CDR3 Id-peptide derived from the V_L_ of the M315 myeloma protein, presented on the MHC class II molecule I-E^d^ (7). Tumor-infiltrating CD11b^+^ cells from TCR-Tg mice that reject MOPC315 tumors induce a dose-dependent cytotoxicity against MOPC315 myeloma cells *in vitro* [(2) and Figure 1A]. In a novel and more sensitive assay, >80% tumor cell death was observed following 4–6 h of co-culture (Figure 1B). To further assay the kinetics of tumor cell death, effector caspase activity was assessed by live imaging in the presence of macrophages, Id-peptide, and Id-specific CD4^+^ T cells. A sharp increase in caspase 3/7 activity was observed after 7–9 h of co-culture in the presence of macrophages and tumor-specific T-cells, whereas co-incubation with macrophages or T cells alone did not cause caspase activation (Figure 1C). Of note, tumor cell killing efficacy and kinetics were comparable to that caused by IFN-γ/LPS-activated macrophages (Figure 1C). Terminal deoxynucleotidyl transferase dUTP nick end labeling (TUNEL) of tumor cells isolated from TCR-Tg and SCID mice on day +10 following s.c. challenge with MOPC315 cells embedded in Matrigel revealed a prominent increase in the number of TUNEL-positive tumor cells in TCR-Tg mice compared to SCID controls (Figure 1D). These results indicate a role for apoptotic cell death in CD4^+^ anti-tumor responses *in vivo*.

To identify the pathway of apoptosis induction, we generated MOPC315 variants overexpressing either the long splice form of c-FLIP (c-FLIP-L) or Bcl-XL (or Bcl-2), thus inhibiting signaling through the extrinsic and intrinsic pathway of apoptosis, respectively. Even though MOPC315 cells showed a modest susceptibility to Fas-mediated killing by co-incubation with the Fas ligand-expressing cell line L5178Y-FasL (Figure 1E), c-FLIP-L-overexpressing tumor cells showed no resistance toward killing by macrophages (Figure 1F). Similar negative results were obtained using MOPC315 cells overexpressing the viral protein CrmA, which has been shown to mediate resistance to apoptotic cell death occurring through the CD95 and TNF pathways (Figure 1E and *data not shown*) (19). In contrast, tumor cells overexpressing either Bcl-XL or Bcl-2 were protected against macrophage-mediated killing (Figure 1F).

To confirm the *in vivo* relevance of these findings, TCR-Tg SCID and SCID mice were challenged with MOPC315 cells overexpressing c-FLIP-L or Bcl-XL. Both cell lines lead to tumor development in SCID mice with kinetics comparable to that of mock-transfected cells (Figures 1G,H), and serum levels of the M315 myeloma protein was comparable upon tumor development (Figure 1I). TCR-Tg SCID mice were protected against challenge with MOPC315-FLIP-L (Figure 1G), whereas injection of MOPC315-BclXL cells lead to tumor development with no delay compared to SCID controls (Figure 1H). In summary, these findings demonstrate that macrophage-induced killing of MOPC315 cells occurs by signaling through the intrinsic pathway of apoptosis.

### Macrophage-Mediated Cytotoxicity Is Spatially Limited and Is Dependent on Short-Lived, Secreted Factors

**Figure 2:**
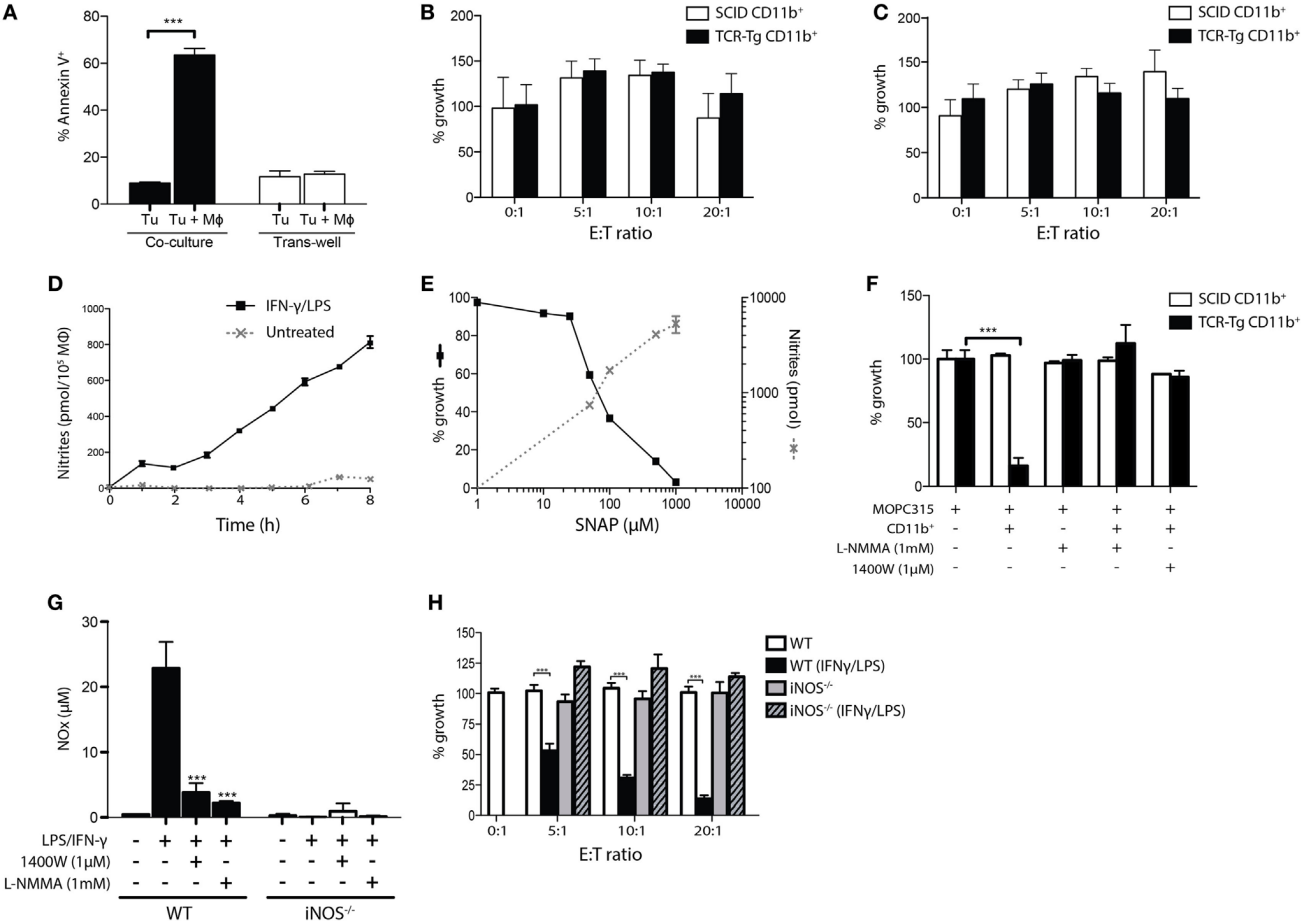
Killing of myeloma cells by activated macrophages is dependent on inducible nitric oxide synthase (iNOS)-mediated release of nitric oxide. **(A)** CD11b^+^ cells were isolated from Matrigels at day +12 following tumor challenge in T cell receptor-transgenic (TCR-Tg) mice, and co-incubated with tumor cells in co-culture or separated by a trans-well membrane at a 20:1 ratio. Surface exposure of Annexin V on tumor cells was determined after 24 h. **(B,C)** Cytotoxicity of glutaraldehyde-fixed Matrigel-derived CD11b^+^ cells **(B)** and culture supernatant isolated after incubation of CD11b^+^ cells **(C)** seeded at various ratios to tumor cells. Cells were incubated for 48 h with the addition of ^3^H-thymidine for the last 18 h of culture. Growth was expressed as percentage counts per minute (cpm) of tumor cells cultured alone. **(D)** Accumulation of nitrites in supernatant of either interferon gamma (IFN-γ)/LPS-treated or untreated peritoneal macrophages was determined at various length of culture using the Griess assay. **(E)** MOPC315 tumor cell growth inhibition induced by the NO donor SNAP, plotted against the nitrite concentration at 4 h. **(F)** Tumor cell growth inhibition assay with CD11b^+^ macrophages in the presence or absence of the NOS inhibitor L-NMMA or the selective iNOS inhibitor 1400W. Tumor-derived CD11b^+^ cells from TCR-Tg or SCID mice at day +12 following challenge were pre-treated for 2 h with the NOS inhibitors before the addition of tumor cells. After the 6 h co-incubation, tumor cells were washed and cultured for another 24 h with the addition of ^3^H-thymidine for the last 18 h of culture (mean ± SD). **(G)** Griess assay showing NO release from *in vitro* cultured peritoneal macrophages from wild-type (WT) or iNOS-deficient (iNOS^−/−^) mice. Macrophages were kept in a resting state or activated with IFN-γ/LPS and cultured in the presence or absence of the iNOS inhibitors 1400W or L-NMMA. Results are shown as mean + SD, with eight replicates per treatment condition. **(H)** Tumor cell growth inhibition assay showing the effects of co-culture of MOPC315 cells with resting or IFN-γ/LPS-activated peritoneal macrophages isolated from WT or iNOS-deficient (iNOS^−/−^) BALB/c mice at the indicated ratios (mean ± SD).

Using trans-well culture assays, we found that cytotoxicity of TAMs was abrogated when cells were separated by a semipermeable filter, suggesting that killing of tumor cells occurs in a cell contact-dependent manner (Figure 2A). However, no cytotoxicity was observed by exposure of tumor cells to glutaraldehyde- or paraformaldehyde-fixed cells (Figure 2B). Incubation of tumor cells with macrophage-conditioned medium failed to induce cytotoxicity (Figure 2C). These results indicate that killing is dependent on living macrophages that secrete cytotoxic factors with a short half-life.

### NO-Derived Metabolites Mediate Macrophage Cytotoxicity Against Tumor Cells

Given the apparent short range and limited half-life of the cytotoxic factor(s) involved in macrophage-mediated killing of tumor cells, we assayed the secretion of RNS by peritoneal macrophages that had been activated with IFN-γ/LPS. Accumulation of NO occurred in a linear manner, with nitrites reaching an accumulated dose of ~6.5 μM after 6 h, corresponding to an NO secretion rate of ~100 pmol/h/10^5^ Mϕ (Figure 2D). A direct cytotoxic effect of NO against MOPC315 cells was demonstrated using the NO donor SNAP. Addition of SNAP to the culture medium induced dose-dependent killing of MOPC315 cells, with an LD_50_ of ~70 μM, corresponding to a nitrite dose of ~800 pmol (Figure 2E). *In vitro* co-culture assays revealed that macrophage-mediated cytotoxicity was completely abrogated by treatment with the iNOS inhibitors L-NG-monomethylarginine (L-NMMA) and 1400W (Figure 2F; *p* < 0.001), which significantly reduced macrophage NO secretion (Figure 2G). Induction of iNOS expression in macrophages upon cognate interaction with Th1 cells is well described, and is thought to involve signaling *via* IFN-γ and TNF alpha released by the CD4^+^ T cell (20). IFN-γ/LPS- or Th1 T cell-primed peritoneal macrophages from iNOS-deficient mice showed undetectable levels of NO secretion (Figure 2G and *data not shown*), confirming that iNOS is responsible for the NO secretion by activated macrophages. To further verify the relevance of RNS in macrophage cytotoxicity against MOPC315, macrophages from iNOS-deficient BALB/c mice were utilized in *in vitro* killing assays. Tumor cell killing by IFN-γ/LPS-activated peritoneal macrophages was completely abrogated in the absence of iNOS (Figure 2H; *p* < 0.001), despite preserved macrophage activation as indicated by increased surface expression of MHC class II and CD86 (*data not shown*).

### CD4^+^ T-Cell Mediated Immunoprotection Is Mediated by iNOS Expression in Tumor-Associated Macrophages

**Figure 3:**
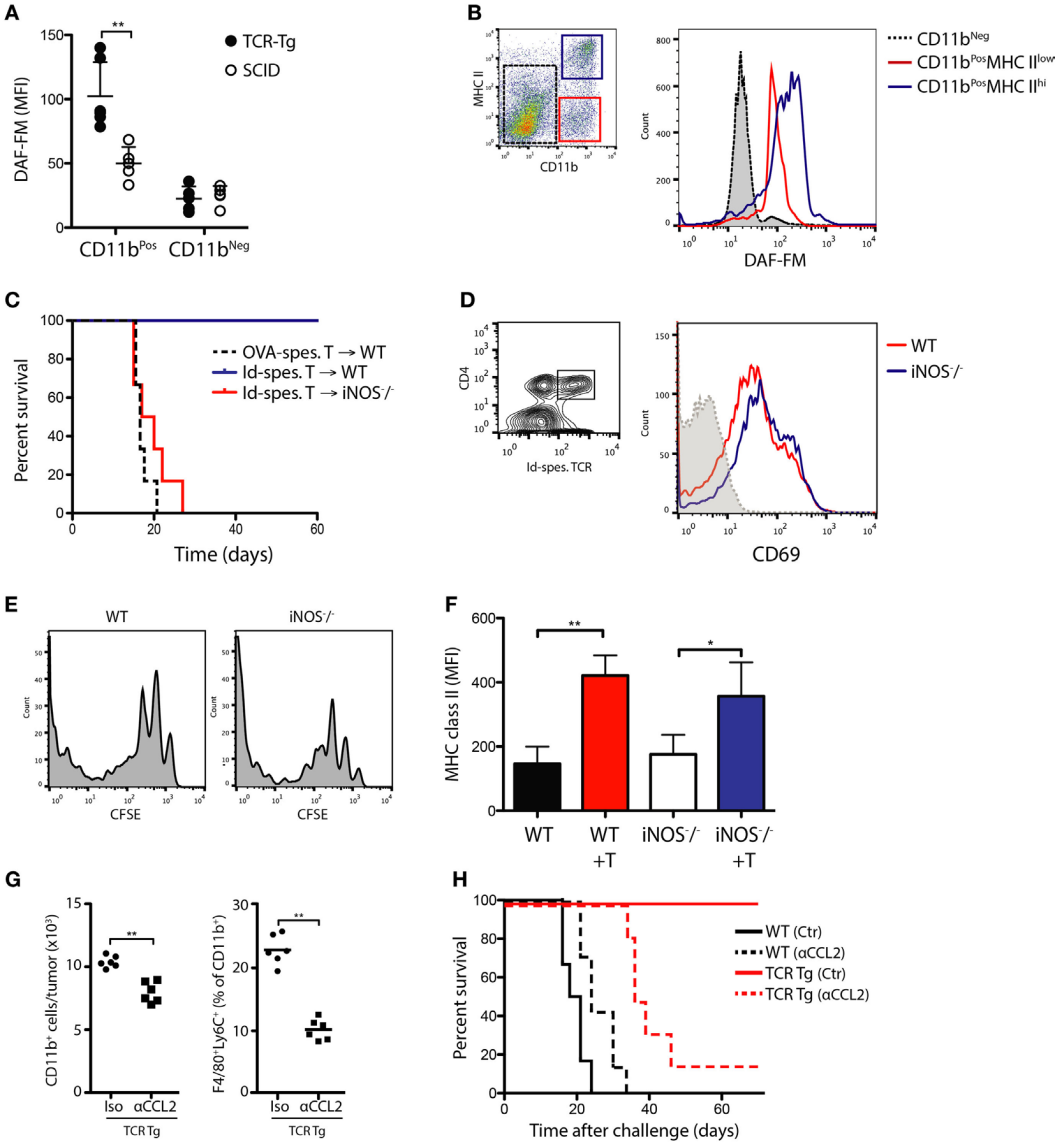
*In vivo* Th1/M1-mediated killing of myeloma cells is mediated by nitric oxide release from tumor-infiltrating macrophages. **(A)** Flow cytometry quantitation of the nitric oxide indicator DAF-FM in CD11b^POS^ and CD11b^NEG^ cells isolated from Matrigels of mice challenged with MOPC315 cells/Matrigel 12 days previously (mean ± SD, *n* = 5/group). **(B)** Distribution of DAF-FM signals in MHC II^HIGH^ and MHC^LOW^ subsets of CD11b^POS^ cells from Matrigel of T cell receptor-transgenic (TCR-Tg) mice. Signal from CD11b^NEG^ cells is shown as a reference. Results are representative of two independent experiments (*n* = 6 per group). **(C)** Survival of MOPC315-challenged wild-type (WT) and iNOS^−/−^ BALB/c mice adoptively transferred with 2 × 10^6^ Id-specific CD4^+^ T cells from TCR-Tg mice or OVA-specific CD4^+^ T cells from DO11.10 mice (*n* = 10–12/group, pooled from two experiments). **(D)** CD69 expression level on Id-specific CD4^+^ T cells in tumor-draining lymph node on day +12 after adoptive T cell transfer in WT and iNOS^−/−^ mice followed by tumor challenge. Results are representative of assays with *n* = 6 mice per group. **(E)** CFSE staining in Id-specific CD4^+^ T cells from lymph nodes of WT or inducible nitric oxide synthase (iNOS)-deficient (iNOS^−/−^) mice at day +12 following s.c. tumor challenge with 1.6 × 10^5^ MOPC315 cells. 2 × 10^6^ CFSE-labeled T cells were adoptively transferred 24 h before tumor challenge. Results are representative of stainings from six mice per treatment group. **(F)** Mean MHC II expression level on CD11b^+^ cells isolated from Matrigels on day +12 following MOPC315 challenge in WT or iNOS^−/−^ BALB/c mice. (+T) indicated adoptive transfer of Id-specific T cells (mean ± SD; *n* = 4–5/group). **(G)** Flow cytometry quantitation of CD11b^+^ cells and macrophages (CD11b^+^F4/80^+^) within the tumor site on day +12 following s.c. challenge with 1 × 10^5^ Matrigel-embedded MOPC315.4 cells in TCR-Tg mice. Mice were treated with antibodies against CCL2 (αCCL2) or polyclonal hamster IgG (Iso). *n* = 5–6 per treatment group. **(H)** Survival of MOPC315-challenged TCR-Tg and WT mice treated with αCCL2 or isotype control mAb. *n* = 5–6 per treatment group.

We have previously shown that successful immunoprotection against MOPC315 is associated with upregulation of M1 macrophage markers, notably iNOS, in TAMs (13). To assay NO formation within tumors, we utilized the fluorescent reagent DAF-FM diacetate to quantify NO production *ex vivo* directly after excision. Tumor cells were suspended in Matrigel and injected s.c. into TCR-Tg and SCID mice, creating a defined tumor bed that could be excised en bloc at various times following challenge. At day +12 post-challenge, a significant increase in NO formation was observed within the incipient tumor site of TCR-Tg mice compared to that of SCID controls (Figure 3A; *p* < 0.01). Importantly, NO production was largely confined to CD11b^+^ macrophages (Figure 3A). In accordance with previous data showing that tumor killing is restricted to CD11b^+^MHC II^high^ cells (16), there was a significantly higher NO production in activated CD11b^+^ cells with high MHC II expression level (Figure 3B).

To directly assess the importance of iNOS for tumor killing *in vivo*, iNOS^−/−^ mice were adoptively transferred with naïve Id-specific T cells and challenged with MOPC315 cells 24 h later. In contrast to WT controls, iNOS-deficient mice showed a complete loss of protection against MOPC315 development (Figure 3C), despite comparable levels of activation of Id-specific CD4^+^ T cells (Figures 3D,E). Of note, tumor-infiltrating CD11b^+^ cells showed comparable expression of MHC class II in the presence of Id-specific CD4^+^ T cells, indicating that cognate interaction of macrophages with T cells was not impaired in the absence of iNOS (Figure 3F). In accordance with the *in vitro* data, these results confirm the essential role of iNOS as a mediator of cytotoxicity in our model.

Previous reports have shown that, in *ex vivo* isolated single cell suspensions from MOPC315 tumors, cytotoxicity is confined to a population of CD11b^+^ cells. To obtain direct evidence of the role of macrophages for *in vivo* killing, we found that the use of a neutralizing mAb against the chemokine (C-C motif) ligand 2 (CCL2) resulted in a selective depletion of CD11b^+^F4/80^+^ cells within the tumor (Figure 3G), and abrogated immunoprotection in TCR-Tg mice (Figure 3H). This provides further confirmation that TAMs are responsible for *in vivo* CD4^+^ T-cell-mediated killing.

### Cytotoxicity Is Mediated by Peroxynitrite Formation Within the Tumor Cells

The toxic effect of NO might be related to the formation of secondary oxidants (peroxynitrite) by reaction with superoxide ions. High levels of peroxynitrite may induce apoptosis (21) through a number of mechanisms [reviewed in Ref. (22)]. Formation of peroxynitrite could occur either extracellularly by reaction with extracellular superoxide of macrophage or tumor cell origin, or within the tumor cells. To approach this issue, a number of experimental strategies were employed.

**Figure 4:**
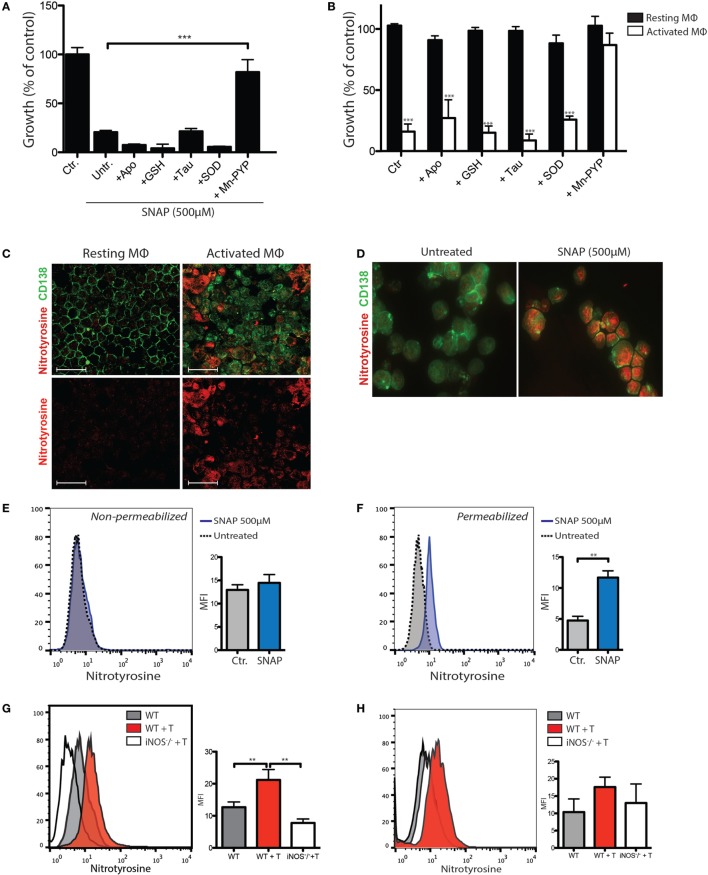
NO-mediated tumor killing is associated with intracellular accumulation of peroxinitrite metabolites. **(A)** Tumor cell growth inhibition assay showing the effects on MOPC315 growth of 4 h incubation with the NO donor SNAP (500 µM) or vehicle (Ctr.). The following ROS/reactive nitrogen species (RNS) inhibitors were added to tumor cells 2 h before the addition of SNAP; the cell-permeable NADPH oxidase inhibitor Apocynin (300 µM), the cell-impermeant superoxide scavenger glutathione (GSH; 2.5 mM), the hypochlorite scavenger Taurine (20 mM), the cell-impermeable superoxide scavenger superoxide dismutase (SOD; 150 U/mL) and the peroxynitrite scavenger MnTE-2-PyP (100 µM). Untreated controls (Untr.) were incubated with DMSO vehicle only. After 4 h incubation with SNAP, cells were washed and cultured for another 24 h with the addition of ^3^H-thymidine for the last 18 h of culture. Growth was expressed as percentage counts per minute (cpm) of tumor cells cultured alone. Results are representative of three independent assays. Results are shown as mean + SD of quadruplicates. **(B)** MOPC315 cells (1 × 10^4^) were added to 2 × 10^5^ resting or interferon gamma (IFN-γ)/LPS-activated peritoneal macrophages that had been pre-incubated with the indicated ROS/RNS inhibitors for 1 h. **(C)** Immunofluorescence staining of cytospin preparations of MOPC315 cells co-incubated with resting or IFN-γ/LPS-activated macrophages. Images are representative of three independent assays. White scale bars indicate a distance of 50 µm. **(D)** Immunofluorescence staining of cytospin preparations of MOPC315 cells incubated 24 h in the presence or absence of the NO donor SNAP (500 µM). Cells were fixed in methanol and stained with anti-nitrotyrosine (red) and anti-CD138 (green). Photographs were taken at 40× magnification with identical exposure settings. **(E,F)** Nitrotyrosine staining in flow cytometry of MOPC315 cells treated with the NO donor SNAP for 4 h. Non-permeabilized **(E)** or fixed/permeabilized **(F)** cells were stained with an anti-nitrotyrosine antibody. Histograms show nitrotyrosine staining in representative samples. Graphs show mean fluorescence intensity (MFI) levels of cells for the various treatment conditions (*n* = 4 per group). **(G,H)** Flow cytometry staining for nitrotyrosine in permeabilized **(G)** or non-permeabilized **(H)** CD138^+^ tumor cells isolated from Matrigels on day +12 following s.c. MOPC315 challenge in wild type (WT + T) or inducible nitric oxide synthase (iNOS)-deficient (iNOS^−/−^ + T) BALB/c mice adoptively transferred with 2 × 10^6^ Id-specific CD4^+^ T cells. BALB/c mice not receiving Id-specific T cells (WT) is shown as a control. Graphed results (right) are shown as mean + SD of MFI (*n* = 6/group). Results are representative of two independent assays.

To assay the contribution of extracellular superoxide, the cell-impermeant superoxide scavenger SOD and small molecular weight inhibitors of NADPH oxidase (Apocynin) were assayed for their ability to inhibit toxicity of the NO donor SNAP (Figure 4A) and macrophage-mediated killing (Figure 4B). To assay the roles of intracellular RNS, the peroxynitrite scavenger MnTE-PyP was tested for its ability to prevent cytotoxicity (Figures 4A,B). Immunostaining for nitrotyrosine, an indicator of peroxynitrite generation (23), confirmed intracellular accumulation of peroxynitrite in MOPC315 cells following exposure to SNAP (Figure 4C). In summary, these results demonstrated that NO-mediated cytotoxicity occurs *via* the formation of peroxynitrite, predominantly by reaction with oxygen radicals generated within the tumor cells (Figures 4A,B). Extracellular superoxide scavengers do not appear to inhibit, but rather increase cytotoxicity (Figures 4A,B), suggesting that macrophage-derived superoxide does not contribute to cytotoxicity against the tumor cells. Immunostaining for nitrosylated proteins revealed a strong increase in intracellular nitrotyrosine accumulation upon co-incubation with activated macrophages (Figure 4C), similar to the result of exposure to SNAP (Figure 4D). Correspondingly, cell surface nitrosylation following treatment with SNAP was minimally increased, whereas intracellular flow cytometry showed a significant accumulation of nitrotyrosine (Figures 4E,F).

To evaluate peroxynitrite formation *in vivo*, WT or iNOS^−/−^ BALB/c mice were adoptively transferred with Id-specific CD4^+^ T cells and challenged with Matrigel-embedded MOPC315 cells 24 h later. Cells were isolated from Matrigels 10 days after adoptive transfer. Residual CD138^+^ tumor cells were assayed for nitrotyrosine by flow cytometry. Increased intracellular nitrotyrosine accumulation in residual tumor cells was observed in WT, but not iNOS-deficient mice after adoptive T cell transfer (Figure 4G). These results are indicative of iNOS-dependent formation of peroxynitrite within tumor cells during killing. A similar trend was seen for surface nitrotyrosine, although the differences were less pronounced and did not reach statistical significance (Figure 4H).

### NO-Induced Killing of Tumor Cells by Macrophages Is Spatially Restricted

**Figure 5:**
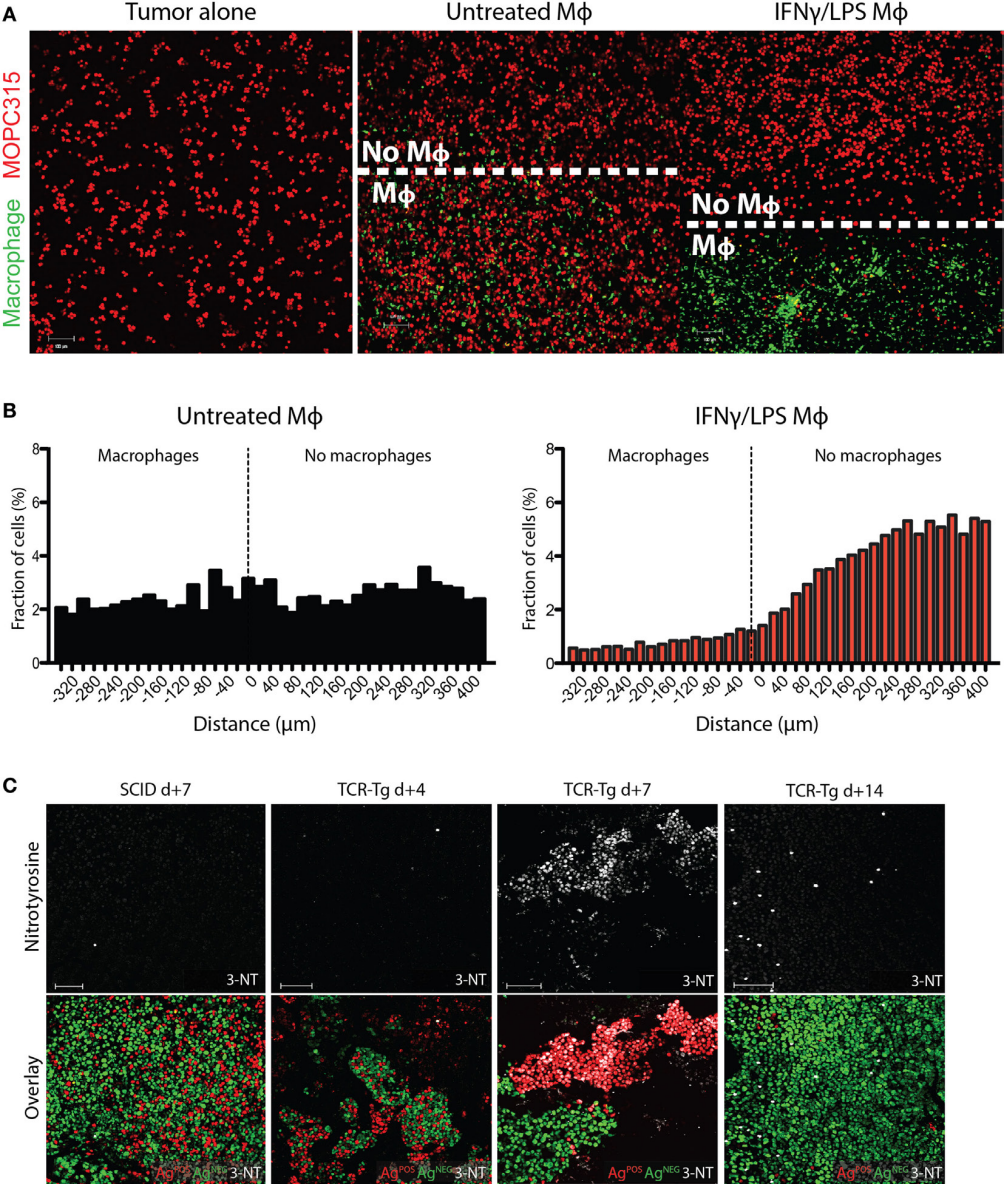
Spatial restrictions of inducible nitric oxide synthase (iNOS)-mediated killing of tumor cells. **(A)** Representative images of *in vitro* Matrigel co-culture assays of peritoneal macrophages (green) and MOPC315 cells (red). Cell-culture insert confining the adherent macrophages to a defined portion of the well were removed after adhesion, prior to co-culture with tumor cells. Images show the border areas in cultures of tumor cells cultured alone or in the presence of untreated or interferon gamma (IFN-γ)/LPS-activated macrophages for 24 h. **(B)** Spatial distribution of tumor cells relative to the front of the macrophage layer in the assay described in **(A)**, expressed as shortest border-border distance (μm). Results were obtained by distribution plotting of 2 × 10^4^ tumor cells per treatment condition, based on three independent assays. Results are expressed as relative frequencies within the range of −340 to +400 μm from the macrophage border. See also Movies S1 and S2 in Supplementary Material. **(C)** Representative immunofluorescence staining for 3-nitrotyrosine (white) of Matrigels on days +4, +7, and +14 following s.c. challenge with 1:1 mixtures of antigen-secreting (Ag^POS^; red) and antigen-negative (Ag^NEG^; green) MOPC315 cells in Id-specific T cell receptor-transgenic (TCR-Tg) SCID and SCID mice. Images were taken at 10× magnification. 100 µm scale bars are shown in white.

The short half-life of RNS would be expected to limit the range of macrophage-mediated cytotoxicity. To get an impression of the range of toxic effects, fluorescently labeled peritoneal macrophages were seeded in chamber slides containing a removable insert, creating a macrophage monolayer covering a defined portion of the slide. Upon removing the insert, the chamber was overlaid with a surplus of MOPC315 tumor cells suspended in liquid Matrigel that gellifies on subsequent culture at 37°C. The distribution of living tumor cells was then determined by confocal microscopy at various time points. After 24 h incubation, only few living tumor cells were seen corresponding to areas containing IFN-γ/LPS-primed macrophages, whereas the number of live tumor cells rapidly increased from a distance of 80 to 120 µm from the edge of the macrophage border (Figures 5A,B). By contrast, non-activated macrophages did not affect the growth of overlying tumor cells (Figures 5A,B). Live cell imaging similarly revealed a non-contact-dependent but spatially restricted killing of tumor cells by activated macrophages (Movies S1 and S2 in Supplementary Material). We thus conclude that the effective range of cytotoxicity of activated macrophages is limited to 80–120 µm.

Spatial constraints of macrophage cytotoxicity may impose a limit on the efficacy of bystander killing of antigen-negative cells within a heterogeneous tumor (16). To further explore this issue, we mixed and embedded differentially labeled antigen-secreting (Ag^POS^) and antigen-negative (Ag^NEG^) MOPC315 variants at a 1:3 ratio in Matrigel, followed by injection s.c. into TCR-Tg and SCID mice. Consistent with previous results, Ag^NEG^ cells were found to accumulate into separate clusters in TCR-Tg mice as time progressed [Figure 5C; (16)]. By contrast, SCID mice showed more interspersed growth of both Ag^POS^ and Ag^NEG^ cells. Ag^POS^ cells disappeared over time, leaving regions populated with predominantly Ag^NEG^ cells (Figure 5C). Immunostaining of tumor sections showed increased nitrotyrosine formation in TCR-Tg mice compared to SCID controls, with a clear accumulation of nitrotyrosine in areas containing Ag^POS^ tumor cells (Figure 5C). After 7 days, Ag^NEG^ cells were found to cluster in regions that had negligible nitrotyrosine staining, and at day 14, only Ag^NEG^ cells were observed, and nitrotyrosine staining intensity was comparable to that of SCID controls (Figure 5C). These results indicate that the failure of bystander killing of Ag^NEG^ cells is due to the limited range of cytotoxicity of the activated (MHC II^HIGH^) macrophages found in regions of the tumor rich in Ag^POS^ cells.

### Activated Macrophages Induce iNOS-Mediated Killing of Human Tumor Cells

*In vitro* studies of iNOS-mediated human macrophage cytotoxicity are complicated by a lack of reproducible methods to elicit strong NO release in *in vitro*-differentiated primary human macrophages, despite ample evidence in support of NO as a cytotoxic mediator of activated macrophages *in vivo*, as previously reviewed (24, 25). These technical challenges complicate direct *in vitro* assessment of macrophage-mediated cytotoxicity using human macrophages. These limitations notwithstanding, to establish the general applicability of iNOS-mediated cytotoxicity as a means of tumor killing, we co-incubated human tumor cell lines with resting or IFN-γ/LPS-activated murine peritoneal macrophages. Cell lines derived from various human malignancies, including pancreatic, colorectal, and prostate cancer, melanoma, and myeloma were all effectively killed by activated macrophages in an iNOS-dependent manner (Table 1), confirming that a wide range of cancer cell types are susceptible to NO-mediated cell death.

**Table 1:** iNOS-dependent killing of human and murine tumor cell lines.

Name	Origin	Resting Mϕ (% growth ± SD)	Activated Mϕ (% growth ± SD)	Activated Mϕ + L-NMMA (% growth ± SD)	Activated Mϕ + 1400W (% growth ± SD)	ATCC #
HT-29	Colorectal adenocarcinoma (h)	69.4 ± 7.9	3.9 ± 1.4	84.9 ± 2.9	73.7 ± 0.4	HTB-38
BxPC3	Pancreatic adenocarcinoma (h)	70.5 ± 3.9	4.3 ± 0.5	73.2 ± 9.7	63.9 ± 3.7	CRL-1687
PANC-1	Pancreatic carcinoma (h)	119.2 ± 10.6	8 ± 1	33.2 ± 4	32 ± 4.8	CRL-1469
H929	Multiple myeloma (h)	101.5 ± 9.6	8.2 ± 4.3	70.7 ± 6.7	71 ± 6	CRL-9068
B16F10	Mouse melanoma (m)	112.5 ± 5.4	0.6 ± 0.3	42.5 ± 4.1	34.8 ± 2.8	CRL-6475
A20	B lymphoma (m)	183 ± 14.6	8.2 ± 3.1	154.6 ± 17.8	154.6 ± 10.1	TIB-208

*Cytotoxicity of resting or IFN-γ/LPS-activated murine peritoneal macrophages in 48 h co-culture assays with tumor cells of human (h) or murine (m) origin, E:T ratio 20:1. Results show % growth compared to tumor cells cultured alone. The iNOS inhibitors L-NMMA (1 mM) and 1400W (1 µM) were added to the culture medium at the start of the experiment where indicated. Results show mean ± SD values from four to eight replicates per treatment condition*.

*iNOS, inducible nitric oxide synthase; ATCC, American Type Culture Collection; IFN-γ, interferon gamma*.

## Discussion

Macrophages may function as potent APCs, and serve as mediators of both innate and adaptive immune responses. Considering their abundance within most tumors, these cells constitute an attractive target of immunotherapeutic interventions. Their potential therapeutic relevance is further emphasized by the growing appreciation of the importance of CD4^+^ T cells in anti-tumor immune responses, not only in murine models (26–28) but also in clinical trials (29–31). Although a number of reports have demonstrated cytotoxic effects of classically activated macrophages *in vitro*, less is known about the mechanisms of macrophage-mediated tumor killing *in vivo*.

The present results identify the molecular mechanisms underlying macrophage-mediated killing of tumor cells in the context of a CD4^+^ Th1 cell response against MHC class II negative multiple myeloma cells. In this model, the anti-tumor immune response is dependent on uptake and presentation of secreted tumor antigen on TAMs (12). Cytotoxicity is mediated by T cell-driven IFN-γ-mediated activation and M1 polarization of macrophages (2). We here show that this interaction results in increased expression of iNOS, and release of nitric oxide from the activated macrophage. Diffusion of NO into surrounding tumor cells leads to accumulation of toxic secondary metabolites, including peroxynitrite, and triggers apoptotic cell death by activation of the intrinsic apoptotic pathway. These findings are in agreement with numerous previous reports demonstrating potent *in vitro* toxicity of NO against tumor cells, and a growth-inhibitory effect of ectopic iNOS expression in tumor cells (32–35). Interestingly, studies of tumor-specific CD8^+^ T cell effector function suggests that induction of NO production by infiltrating macrophages might similarly play an important role in CD8^+^ T cell responses against cancer (36).

In a previous report, genetic ablation of iNOS was shown to impair immunoprotection after whole-cell vaccination in a B16 melanoma model (37). Although the mechanistic basis for this observation was not addressed, it was shown that the presence of CD4^+^ T cells was required for the appearance of iNOS-expressing macrophages within the tumor bed following vaccination (37). These previous results indicate that the presently described mechanisms of Th1/M1-mediated elimination of tumor cell may also be relevant in other models of anti-tumor immune responses. Moreover, it is also possible that indirect, macrophage-mediated responses may contribute to the cytotoxicity against MHC class II positive tumors as an adjunct to direct cytotoxic effects of CD4^+^ T cells on such tumor cells. Indeed, emerging data from our group suggest that tumor-cell-intrinsic MHC II expression is dispensable for *in vitro* CD4^+^ T-cell-mediated killing in the B16 melanoma and A20 lymphoma (38). The importance of macrophages and iNOS as effectors in these, and other, model systems is the subject of ongoing investigation. Our assays using human and murine tumor cell lines of various origins, including pancreatic, colorectal, and prostate adenocarcinoma, melanoma, and myeloma, point to iNOS-mediated macrophage cytotoxicity as a broadly applicable mechanism of tumor killing.

The toxic effects of nitric oxide are mediated by diffusion into surrounding tumor cells, and further reaction with intracellular metabolites such as superoxide to form toxic mediators such as peroxynitrite within the tumor cell. The immunoprotection offered by CD4^+^ T cells is completely dependent on iNOS activity, and is prevented by inhibition of iNOS activity or by scavenging of peroxinitrite. Interestingly, perxynitrite-mediated apoptosis induction has been reported to occur through a process that is inhibited by Bcl-2/Bcl-XL (39, 40), in agreement with our findings that overexpression of these anti-apoptotic proteins abrogates macrophages-mediated cytotoxicity. Collectively, these findings are consistent with a role of peroxynitrite as a major effector in inducing tumor cell death upon exposure to Th1-activated macrophages. As previously mentioned, induction of NO release by *in vitro*-differentiated human macrophages has proven difficult, which has lead to some controversy regarding the importance of iNOS-derived NO in human macrophages, reviewed in Ref. (24, 25). A number of reports have documented iNOS-dependent macrophage-derived nitric oxide as a cytotoxic mediator in infectious diseases, including Leishmaniasis (41) and tuberculosis (42), as well as in sterile inflammatory conditions (43, 44), suggesting that the lack of *in vitro* NO production represents a technical artifact of current *in vitro* culture systems. It is conceivable that these limitations might be overcome through the use of *ex vivo* isolated, patient-derived TAMs in co-culture assays. These issues are being addressed through ongoing studies, and may hopefully shed light on the relevance of NO as a cytotoxic mediator in human cancer.

The non-discriminant nature of macrophage cytotoxicity creates a potential for bystander damage to surrounding tissue during inflammatory processes. Using a three-dimensional Matrigel culture system, we demonstrate that induction of apoptosis by activated macrophages is limited to tumor cells within a ~80–120 μm range. While compromising the efficacy of cytotoxicity in a tumor setting (16), such a spatial restriction might be an important means of limiting damage to surrounding tissue by innate cells. These observations may therefore be of relevance to the understanding of tissue homeostasis in the context of a wide variety of inflammatory stimuli.

We have previously reported a lack of bystander killing of antigen-negative tumor cells in our TCR-Tg mice due to a failure to induce macrophage activation in areas of the tumor bed dominated by antigen-negative tumor cells (16). Nitrotyrosine staining of incipient tumors containing mixed populations of antigen-secreting and non-producing MOPC315 cells shows that nitrosylation is more pronounced in areas surrounding antigen-secreting tumor cells. Upon outgrowth of antigen-negative cells, nitrosylation is sparse and at levels comparable to SCID controls. These results thus provide a mechanistic basis for the lack of bystander killing that is defined by the limited range of toxicity of secreted NO.

In summary, our findings demonstrate that CD4^+^ T cells reactive against a secreted tumor antigen mediate elimination of tumor cells by inducing ROS/RNS signaling by TAMs. Activation of macrophages leads to the generation of peroxynitrite within cells in their immediate surroundings, which induces apoptotic cell death of neighboring tumor cells. The abundance of macrophages within tumors points to induction of ROS/RNS signaling as a means to induce potent therapeutic anti-tumor responses, not only in the course of adaptive immune responses but also as a response to other interventions that induce inflammation within the tumor bed, including cytotoxic agents.

## Ethics Statement

All animal experiments were approved by the Norwegian Animal Research Authority (Mattilsynet), and performed in accordance with institutional and Federation of European Laboratory Animal Science Associations (FELASA) guidelines.

## Author Contributions

MF and OH contributed to the design of the study, performed experiments and data analysis, created figures, and revised the manuscript. FS contributed to the design of the study and performed experiments. BB contributed conception and design of the study, analyzed data, and revised the manuscript. AT contributed conception and design of the study, performed experiments and data analysis, wrote and revised the manuscript. All authors contributed to manuscript revision, read and approved the submitted version.

## Conflict of Interest Statement

The authors declare that the research was conducted in the absence of any commercial or financial relationships that could be construed as a potential conflict of interest.

## Supplementary Material

movie S1 Timelapse microscopy of co-cultures of CFSE-labeled non-activated peritoneal macrophages (green; lower part of field) and MOPC315 cells (round suspension cells). Propidium iodide (red signal) indicates dying cells. Images were taken at 10 minute intervals. Video is representative of three independent assays with multiple assay points per experiments.

movie S2 Timelapse microscopy of co-cultures of CFSE-labeled, IFN-γ/LPS-activated peritoneal macrophages (green, lower part of field) and MOPC315 cells (round suspension cells). Propidium iodide (red signal) indicates dying cells. Images were taken at 10 minute intervals. Video is representative of three independent assays with multiple assay points per experiment.
